# Dendritogenic Potential of the Ethanol Extract from *Lippia alba* Leaves in Rat Cortical Neurons

**DOI:** 10.3390/molecules28186666

**Published:** 2023-09-16

**Authors:** María Marcela Velásquez, María Claudia Lattig, Luis Carlos Chitiva, Geison M. Costa, Jhon Jairo Sutachan, Sonia Luz Albarracin

**Affiliations:** 1 Instituto de Genética Humana, Facultad de Medicina, Pontifica Universidad Javeriana, Bogotá 110911, Colombia; 2 Facultad de Ciencias, Universidad de los Andes, Bogotá 110911, Colombia; 3 Departamento de Química, Pontificia Universidad Javeriana, Bogotá 110911, Colombia; 4 Departamento de Nutrición y Bioquímica, Pontificia Universidad Javeriana, Bogotá 110911, Colombia

**Keywords:** structural plasticity, dendritic complexity, neurotrophins, PI3Ks, MAPKs, flavonoids, terpenes, *Lippia alba*, neuropsychiatric disorders

## Abstract

A reduced dendritic complexity, especially in regions such as the hippocampus and the prefrontal cortex, has been linked to the pathophysiology of some neuropsychiatric disorders, in which synaptic plasticity and functions such as emotional and cognitive processing are compromised. For this reason, the identification of new therapeutic strategies would be enriched by the search for metabolites that promote structural plasticity. The present study evaluated the dendritogenic potential of the ethanol extract of *Lippia alba*, an aromatic plant rich in flavonoids and terpenes, which has been widely used in traditional medicine for its presumed analgesic, anxiolytic, and antidepressant potential. An in vitro model of rat cortical neurons was used to determine the kinetics of the plant’s effect at different time intervals. Changes in morphological parameters of the neurons were determined, as well as the dendritic complexity, by Sholl analysis. The extract promotes the outgrowth of dendritic branching in a rapid and sustained fashion, without being cytotoxic to the cells. We found that this effect could be mediated by the phosphatidylinositol 3-kinase pathway, which is involved in mechanisms of neuronal plasticity, differentiation, and survival. The evidence presented in this study provides a basis for further research that, through in vivo models, can delve into the plant’s therapeutic potential.

## 1. Introduction

Dendritic arborization is a relevant morphological factor that influences the identity and number of presynaptic stimuli that neurons can integrate, thus affecting their overall functionality [1]. Changes in dendritic complexity are decisive for neuronal plasticity dynamics in the central nervous system [2,3]. Accordingly, it has been suggested that irregularities in the formation of spines and dendritic branches, which are the basis of the postsynaptic excitatory machinery, are related to the pathophysiology of multiple neuropsychiatric disorders [4,5]. Specifically, morphological abnormalities of the dendritic tree have been identified in conditions such as autism spectrum disorder, Alzheimer’s disease, schizophrenia, Down syndrome, fragile X syndrome, and Rett syndrome [6,7]. Likewise, considering that dendritic complexity is susceptible to responding to intrinsic and extrinsic factors, such as trauma or stress, it has been related to cognitive and mood disorders, including anxiety and major depression [8,9]. In this regard, chronic stress has been reported to induce neuro-histological changes, especially in regions such as the hippocampus and prefrontal cortex, significantly reducing dendritic arborization and, thus, affecting the glutamatergic neurotransmission system [8,10].

Such changes in synaptic plasticity, which imply irregularities in emotional processing and impairment of cognitive functions [11,12], could emerge because of disruptions in the expression of neurotrophins, such as the brain-derived neurotrophic factor (BDNF), whose activation cascades regulate neuronal survival processes and signal transduction [13]. BDNF is involved in the signaling pathways of mitogen-activated protein kinases (MAPKs) and phosphatidylinositol 3-kinases (PI3Ks), which play a crucial role in both neuronal survival and outgrowth of dendrites [13,14]. It has been suggested that these signaling pathways form an intracellular network for the modulation of neuroplasticity, suggesting that their disruption might be involved in the development of different psychiatric and neurodegenerative disorders [15,16]. The identification of components that have the potential to promote neuronal survival and increase dendritic complexity, particularly at the prefrontal cortex, would represent a real advance in the development of effective treatments for a board spectrum of neuropsychiatric and neurodegenerative conditions [17,18].

These compounds that enhance cortical plasticity, called psychoplastogens [19,20,21], include natural metabolites very promising for treating some mood disorders. This is the case of psilocybin (precursor of psilocin, 4-hydroxy-dimethyltryptamine), a hallucinogenic alkaloid naturally synthesized by some species of mushrooms of the *Psilocybe* genus [22]. Several recent studies have shown this secondary metabolite’s great potential for treating a wide spectrum of mood disorders, including treatment-resistant depression [19,20,23,24,25]. Likewise, there is experimental evidence for the dendritogenic effect of the alkaloid [21], as well as for its few side effects [26], and it has been well established that it does not imply a risk of addiction [27,28]. However, although in several countries the legal restrictions of using this type of psychotropic substances for research purposes have been gradually relaxed [29], they remain a significant limitation in other regions (such as Colombia). This situation, added to the growing interest in identifying new components that can exert effects like those of classic psychedelics without being hallucinogenic, encourages the exploration of other natural metabolites.

The present study determined the dendritogenic potential of the ethanol extract from *Lippia alba* leaves. This is an aromatic plant (Verbenaceae) of wide distribution that has been used in traditional medicine in different regions of Latin America, to which analgesic, anxiolytic, and antidepressant properties are attributed [30,31]. Its effects, and those of other species of the same genus, have been evaluated, especially in animal models and in in vitro conditions, and have positioned this type of plant as a biological resource worthy of exploration in a therapeutical context.

## 2. Results

### 2.1. Analysis of Secondary Metabolites in Lippia alba Leaves Extract

The phytochemical analysis qualitatively determined different types of secondary metabolites present in *Lippia alba*. The results showed the presence of flavonoids, phenolic compounds, and tannins in the ethanolic extract of leaves. Steroid-type compounds, coumarins, and alkaloids were not detected (Table 1).

**Figure 1:**
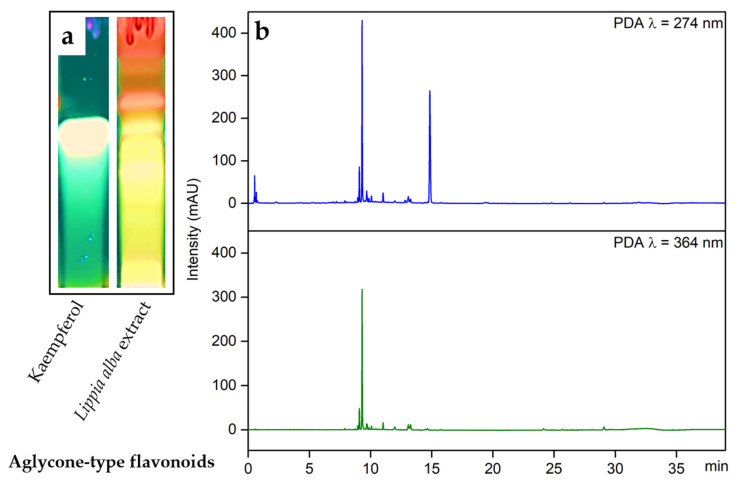
Chromatographic characterization of the *Lippia alba* extract. (**a**) HPTLC was used to visualize flavonoids, silica gel F254 plates were used with a mobile phase of chloroform:methanol (90:10), and detection was performed using natural reagent (NP) and UV light at 366 nm, Kaempferol was used as the standard; (**b**) UPLC chromatograms for the ethanolic extract of *Lippia alba* leaves at 274 and 364 nm.

Through HPTLC screening, the chromatographic profile of the *Lippia alba* extract was determined and compared with the respective standards for flavonoids, steroids, phenolic compounds, alkaloids, and coumarins. Although some types of compounds were not reported for the leaves of this species, some preliminary tests were carried out to determine the possible presence of coumarins and alkaloids. The presence of flavonoids was confirmed by the characteristic fluorescent bands of different colors when derivatization was performed with the natural products reagent (NP) and observed at a wavelength of 366 nm. The UPLC-DAD analysis allowed for the confirmation of the presence of most compounds, mainly corresponding to flavonoids, to be determined, considering the two absorption bands of band A at 240 to 285 nm and band B at 300 to 560 nm (Figure 1).

### 2.2. Lippia alba Extract Increases the Dendritic Complexity of Cortical Neurons

**Figure 2:**
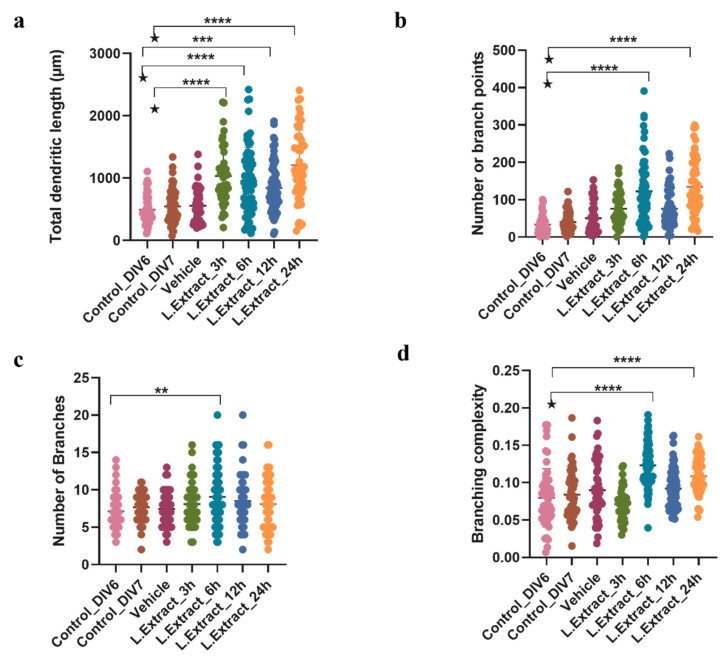
Effect of *Lippia alba* leaf extract on the morphology of cortical neurons. The quantification of changes in the morphological parameters of neurons was performed with the WIS-Neuromath software. (**a**) Total length of dendrites; (**b**) number of branching points; (**c**) number of branches; (**d**) dendritic complexity. Control_DIV6: untreated DIV6 control neurons; Control_DIV7: untreated DIV7 control neurons; vehicle: neurons treated with DMSO (0.2%); L. Extract: neurons treated with extract of *Lippia alba* leaves during specific time intervals (3 h, 6 h, 12 h, 24 h). ★ Indicates that the specific treatment condition differed significantly from Control_DIV6 and Control_DIV7. ** *p* < 0.01, *** *p* < 0.001, **** *p* < 0.0001, compared to controls or the vehicle.

The evaluation of the kinetics of the effect of *Lippia alba* extract (0.01 μg/mL) on the structural plasticity of cortical neurons showed significant morphological changes in the treated neurons at different time intervals (3 h, 6 h, 12 h, and 24 h) compared to the control (untreated neurons, both DIV6 and DIV7) and with the vehicle (DMSO at 0.2%). This effect was reflected in an increased value for several morphological parameters, such as the total length of dendrites [F (6, 388) = 23.38, *p* < 0.0001], the number of branching points [F (6, 378) = 28.16, *p* < 0.0001], the number of branches [F (6, 393) = 3.079, *p* = 0.0059], and the dendritic complexity [F (6, 390) = 19.87, *p* < 0.0001]. The difference was most noticeable 6 h after the application of the extract (Figure 2). Specifically, the one-way ANOVA (95% CI) showed that the neurons treated with the extract during this time interval differed significantly from controls and the vehicle in the number of branch points, their complexity, and the total length of the dendrites (*p* < 0.0001).

**Figure 3:**
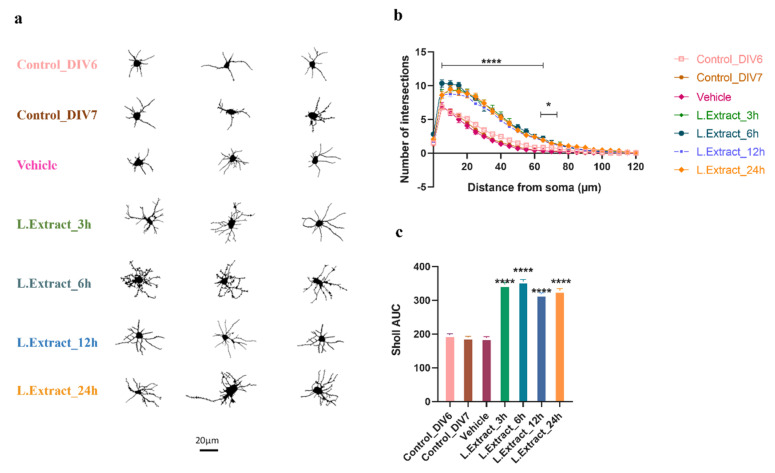
*Lippia alba* extract increased dendritic arborization on cortical neurons. The quantification of changes in the morphological parameters of neurons was performed with Sholl analysis. (**a**) Images obtained from neurons representative of each condition, (**b**) increased dendritic complexity of neurons treated with the extract vs. controls (horizontal lines denote regions in which treatments significantly increased dendritic complexity compared to controls), (**c**) area under the curve (AUC) of Sholl analysis. Control_DIV6: untreated DIV6 control neurons, Control_DIV7: untreated DIV7 control neurons; Vehicle: neurons treated with DMSO (0.2%); L. Extract: neurons treated with extract of *Lippia alba* leaves during specific time intervals (3 h, 6 h, 12 h, 24 h). * *p* < 0.05, **** *p* < 0.0001, compared to controls or the vehicle.

Sholl analysis revealed that the *Lippia alba* extract increased the number of intersections and dendritic complexity in all time intervals, with a more noticeable effect at 6 h (Figure 3). The increase in dendritic complexity was observed in both the proximal (5 to 60 μm of the soma) and distal (61 to 75 μm of the soma) levels, although in the latter case, the effect was only evident for the neurons treated for 6 h. The two-way ANOVA (95% CI) showed that the dendritogenic effect was induced at 3 h and maintained during the 24 h treatment with the *Lippia alba* extract (*p* < 0.0001) and that there was a significant interaction (*p* < 0.0001) between the time intervals of treatment and the distance from the soma.

### 2.3. Specific Inhibitors of ERK1/ERK2 and AKT Do Not Affect the Viability of Neurons after 6 h of Treatment

**Figure 4:**
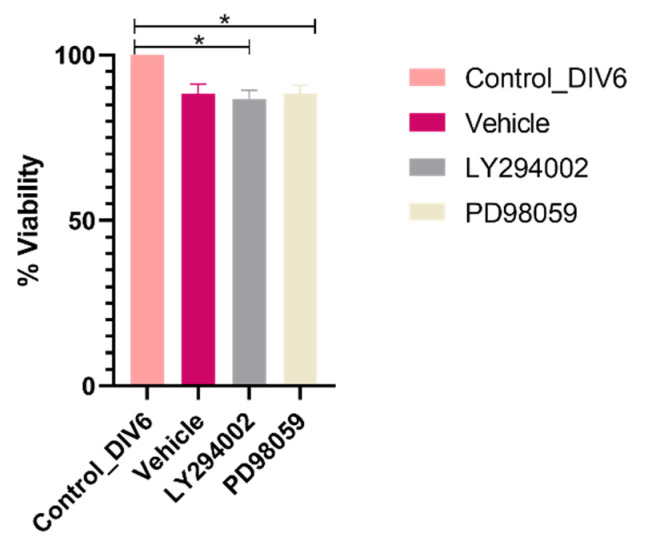
Effect of specific ERK1/ERK2 and AKT inhibitors on the viability of cortical neurons. Control_DIV6: untreated DIV6 control neurons; Vehicle: neurons treated with DMSO (0.2%); LY294002: neurons treated with ERK1/ERK2 inhibitor −10 µM; PD98059: neurons treated with AKT inhibitor −10 µM. Cell viability was calculated as the absorbance ratio of the neurons treated with each inhibitor/absorbance of the control neurons. * *p* < 0.05, compared to the control or the vehicle.

It was necessary to determine whether LY294002 and PD98059 inhibitors affected the viability of the neurons after 6 h of treatment, given that, at this time, the increase in dendritic complexity was induced by the plant extract both at the proximal and distal levels. The MTT reduction viability assay results showed that neither inhibitor decreased neuron viability by more than 10% (Figure 4).

### 2.4. PI3K Pathway Is Involved in the Dendritogenic Effect of Lippia alba Extract on Cortical Neurons

**Figure 5:**
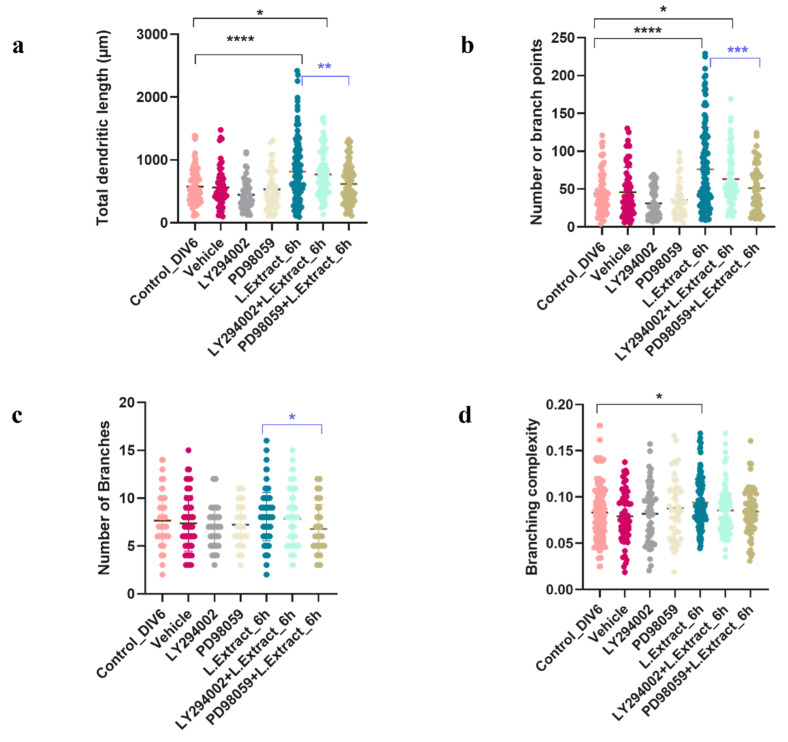
Effect of specific ERK1/ERK2 and AKT inhibitors on the morphology of cortical neurons. The quantification of changes in the morphological parameters of neurons was performed with the WIS-Neuromath software. (**a**) Total length of dendrites; (**b**) number of branching points; (**c**) number of branches; (**d**) dendritic complexity. Control_DIV6: untreated DIV6 control neurons; Vehicle: neurons treated with DMSO (0.2%); LY294002: neurons treated with ERK1/ERK2 inhibitor −10 µM; PD98059: neurons treated with AKT inhibitor −10 µM; L. Extract_6 h: neurons treated with extract of *Lippia alba* leaves for 6 h; LY294002 + L. Extract_6 h: neurons pretreated for 1 h with the ERK1/ERK2 inhibitor and then treated with a mix of inhibitor + L. Extract for 6 h; PD98059 + L. Extract_6 h: neurons pretreated for 1 h with the AKT inhibitor and then treated with a mix of inhibitor + L. extract for 6 h. Black bars and asterisks indicate significant differences from the control; the blue bars and asterisks indicate significant differences from the cells treated with the extract alone. * *p* < 0.05, ** *p* < 0.01, *** *p* < 0.001, **** *p* < 0.0001, compared to the control or the vehicle.

To determine whether the dendritogenic effect induced by the *Lippia alba* extract would be mediated by the signaling pathways of MAPKs and/or PI3Ks, the neurons were pretreated with the specific inhibitor for 1 h and then cotreated with a mixture of the inhibitor (10 μM) + extract (0.01 μg/mL) for 6 h. The one-way ANOVA (95% CI) followed by Tukey’s test showed that the neurons pretreated with LY294002-10 μM inhibitor and then cotreated with the *Lippia alba* extract continued to exhibit changes, compared to controls and the vehicle, in morphological variables, such as the total length of dendrites (*p* = 0.0267) and the number of branching points (*p* = 0.0464). In contrast, there were no significant changes from controls or the vehicle in those cells that received the extract after being pretreated with PD98059-10 mM (Figure 5) for parameters such as the number of branches (*p* = 0.3913), the total length of dendrites (*p* = 0.9931), the number of branching points (*p* = 0.9084), or the branching complexity (*p* > 0.9999).

**Figure 6:**
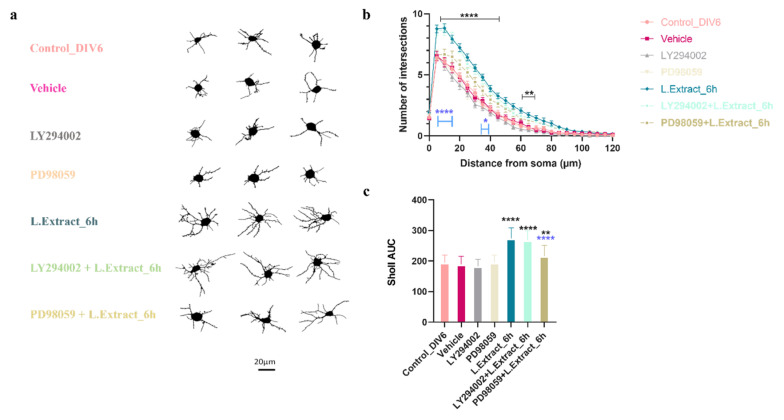
The PI3K pathway is involved in the dendritogenic effect of *Lippia alba* extract on cortical neurons. The quantification of changes in the morphological parameters of neurons was performed with Sholl analysis. (**a**) Images obtained from neurons representative of each condition, (**b**) increased dendritic complexity of neurons treated only with extract of *Lippia alba* and those treated with a mix of LY294002 inhibitor and L. Extract, (**c**) area under the curve (AUC) from Sholl analysis. Control_DIV6: untreated DIV6 control neurons; Vehicle: neurons treated with DMSO (0.2%); LY294002: neurons treated with ERK1/ERK2 inhibitor 10 µM; PD98059: neurons treated with AKT inhibitor 10 µM; L. Extract_6 h: neurons treated with extract of *Lippia alba* leaves for 6 h; LY294002 + L. Extract_6 h: neurons pretreated for 1 h with the ERK1/ERK2 inhibitor and then treated with a mix of inhibitor + L. Extract for 6 h; PD98059 + L. Extract_6 h: neurons pretreated for 1 h with the AKT inhibitor and then treated with a mix of inhibitor + L. extract for 6 h. Black lines and asterisks denote regions where treatments significantly increased dendritic complexity compared to controls; blue lines and asterisks denote regions where pretreatment with PD98059 inhibitor reduced dendritic complexity compared to neurons treated with the extract alone, * *p* < 0.05, ** *p* < 0.01, **** *p* < 0.0001, compared to the control or the vehicle.

The one-way ANOVA (CI 95%) performed for the results of the AUC from Sholl analysis showed that the neurons co-treated with the LY294002 inhibitor + *Lippia alba* extract did not differ in dendritic complexity from the neurons treated only with the plant extract (*p* = 0.9315). However, co-treatment with PD98059 inhibitor + *Lippia alba* extract reduced the dendritic complexity of the neurons compared to the treatment with the plant extract alone (*p* < 0.0001). These results suggest that the PI3K pathway would be involved in the dendritogenic effect of *Lippia alba* extract on cortical neurons (Figure 6).

## 3. Discussion

An altered dendritic arborization has been linked to neurodevelopmental disorders, cognitive impairment conditions, and stress-related psychopathologies [4,6]. Specifically, reduced dendritic complexity in the hippocampus and prefrontal cortex affects neural plasticity in circuits implicated in memory consolidation and emotional processing [5,32,33,34]. It has even been reported that some genetic variants associated with neuropathologies code for essential proteins for postsynaptic excitatory activity [5], for which dendritic branching is essential [35]. In this scenario, it has been suggested that molecules that promote dendritogenesis could be considered therapeutic alternatives for neuropsychiatric disorders [36]. Some classic psychedelics (including psilocybin) are effective psychoplastogens [23,37]. However, in some countries, the possibility of research with such substances (which has been widely stigmatized) has motivated the exploration of non-hallucinogenic analogs [21].

This study provides evidence of the ability of the ethanol extract from *Lippia alba* (Miller) leaves to promote structural plasticity in an in vitro model of rat cortical neurons. This aromatic plant, commonly known as “soon relief”, has been widely used in traditional medicine for treating multiple physical ailments and mood disorders [38,39]. In a mouse model of depression induced by corticosterone, it was found that the extract of a plant of the same genus—*Lippia sidoides*—reversed the symptoms of anhedonia in the animals and its effect was comparable to that produced by the antidepressant fluoxetine [40]. Other studies have focused on evaluating this plant’s antinociceptive, anti-inflammatory, and sedative effects [31,39,41]. The present research found that the extract produces rapid (from 3 h) and sustained (up to 24 h) changes in the morphology of cortical neurons and increases dendritic arborization, even at a low concentration of 0.01 μg/mL. This finding would suggest the plant’s therapeutic potential, considering its role as a psychoplastogen.

Although the chemical composition of essential oils and plant extracts of the genus *Lippia* is variable, high levels of terpenoids and phenylpropanoids have been reported for its species and would be responsible for their multiple effects on the central nervous system [39]. For *Lippia alba*, the main components are citral, thymol, myrcene, carvone, and limonene [42,43]. Different flavonoids have also been found in all chemotypes of *Lippia*, including catechin, apigenin, luteolin, rutin, quercetin, and naringenin [44,45]. The pharmacological properties of both terpenoids and flavonoids have been well documented, and many of them are even found in different types of drugs [46,47,48]. The citral terpenoid, for example, would be essential for the sedative, antinociceptive, and anticonvulsant properties of the plant, acting on the GABAergic system and blocking different ion channels in neuronal membranes [49,50].

The dendritogenic effect reported here could be related to the presence of flavonoids in the extract. These polyphenolic compounds, known to stimulate neurogenesis in the hippocampus [51] or reduce oxidative stress in the brain [52], can activate signaling pathways that control synaptic plasticity [53]. Some flavonoids can alter the phosphorylation status of extracellular signal-regulated kinases (ERK1/ERK2) and protein-kinases B (AKT), activating MAPK and PI3K signaling pathways, respectively [54]. The activation of these two pathways impacts the CREB (cAMP response element) transcription factor, which has binding sites in promoter regions of multiple genes associated with neuronal differentiation, survival, and plasticity [13]. Other flavonoids, such as dihydroxyflavone (present in *Lippia sidoides*), would act as direct agonists of tropomyosin B kinase receptors (TrkB), which are specific to some neurotrophins, such as BDNF [55]. Phosphorylation of TrkB receptors, both pre-and postsynaptically, activates MAPK and PI3K pathways [56]. Other polyphenols enhance BDFN expression in both in vitro and in vivo models [55].

These findings on flavonoid properties are consistent with the dendritogenic potential reported in this study and would confirm that a neurotrophin-mediated mechanism would be responsible for such an effect. Even so, it is essential to note that our results suggest that the PI3K pathway, and not the MAPK, is involved in increasing dendritic complexity. These results are consistent with a study that reported that the effect of BDNF on neurite growth in ganglion neurons of the cochlea would be mediated by the PI3K/AKT pathway and not by MEK/ERK [57]. Similarly, another study concluded that atorvastatin potentiates the growth of neurites in cortical neurons through the phosphorylation of AKT [58]. It has also been shown that the activation of PI3K underlies the increase in the complexity of the dendritic tree in an in vitro model of hippocampal neurons [59]. This evidence emphasizes the role of protein kinases in regulating dendritic growth and, therefore, in structural plasticity [60]. They also show that the independent and joint action mechanisms of the MAPK and PI3K pathways are complex; therefore, evaluating their activation in other tissues and contexts of chronic or acute exposure to treatment would be necessary.

The present study highlights the relevance of traditional medicine by offering evidence of the ability of *Lippia alba* extract to act as a promoting agent of dendritogenesis, a desirable effect in the clinical context for treating some psychiatric disorders. Further studies of in vivo models, considering the acute and chronic effects of the extract, will be crucial to assess the behavioral implications of the plant’s morphological changes and determine its therapeutic potential.

## 4. Materials and Methods

### 4.1. Plant Material and Extract Preparation

Leaves of *Lippia alba* (Mill.) N.E.Br. ex Britton and P.Wilson were collected in Tolima, Colombia (GPS 4°55′0″ N, 74°53′0″ W) and identified by Néstor García at the Herbarium of Pontificia Universidad Javeriana (HPUJ), and were deposited with voucher specimen number HPUJ-30554. The collection was performed under the permit of the Contract for Access to Genetic Resources and Derived Products (Contract No. 212/RGE 0287-6) granted by the Ministry of the Environment and Sustainable Development to the Pontificia Universidad Javeriana (PUJ) and the Colombia Científica/GAT Program. The plant material was dried in an oven with circulating air at 35 °C, for 96 h, followed by grinding in a blade mill. The dried and ground plant material was extracted via percolation with 96% ethanol in a 1:10 (*w*/*v*) ratio at room temperature, protected from light, in 4 cycles of 24 h each with solvent changes. The extracts from the different cycles were combined and concentrated and the ethanol residues were fully removed under reduced pressure via rotary evaporation at a temperature of 35 °C. They were then stored at room temperature in labeled amber bottles.

### 4.2. Chemical Composition Analysis

The chemical composition analysis was carried out following the methodology implemented by Sanabria [61]. This analysis included various chemical tests performed in test tubes to qualitatively determine the presence of several groups of secondary metabolites, such as steroids, flavonoids, phenolic compounds, coumarins, alkaloids, and tannins. For each group of metabolites, the corresponding standards (lupeol, kaempferol, tannic acid, coumarin, theobromine, and gallic acid) were used to compare the results and determine each test’s positive or negative presence.

The HPTLC analysis was performed using a CAMAG^®^ system consisting of an autosampler (ATS 4), a developer (ADC), a derivatized (DV), a development chamber, a visualization chamber, and VisionCATS software (v. 3.0). For the extract, a 10 µL solution at a concentration of 10 mg/mL in EtOH was applied as a band on Merck^®^ HPTLC Silica gel 60 F254 plates (20 × 10 cm), which were eluted with different solvent systems based on the metabolite group observed in the previous assay. The developer used for flavonoids was NP-PEG Natural Reagent.

The UPLC analysis was performed using an Acquity H Class UPLC Waters^®^ system equipped with an Acquity photodiode array detector (PDA), quaternary pump, degasser, and autosampler. Empower^®^ 3 software was used to process the data. We used a Phenomenex^®^ Kinetex EVO C18 column (100 × 2.1 mm, 2.6 µm, 100 Å) at 30 °C. The elution gradient was achieved using 0.1% formic acid in water (solvent A) and acetonitrile (solvent B) as follows: 0 to 3 min, 3% B; 3 to 30 min, 3 to 97% B; 30 to 32 min, 97% B; 32 to 35 min, 97 to 3% B; and 35 to 40 min, 3% B, with a run flow rate of 400 μL/min and an injection volume of 2 µL. We detected the samples at 274 nm and 364 nm.

### 4.3. Animals

Embryonic day 18 (E18) Wistar rats were provided by the Unidad de Biología Comparativa (UBC) of the Pontificia Universidad Javeriana, where authorized personnel with due training performed the euthanasia procedure within a carbon dioxide chamber.

### 4.4. Primary Culture of Cortical Neurons

Primary cortical cultures were established from the tissue of Wistar rat embryos from day 18 (E18). The day before, poly-D-lysine (PDL, Sigma-Aldrich, St. Louis, MI, USA), diluted in deionized water (ddH_2_O), was added to the plates of 96 or 24 wells (according to the protocol). Next, all plates were washed with PBS 1X and ddH_2_O and properly dried. The embryos were extracted by cesarean section once the pregnant rat was euthanized in a carbon dioxide (CO_2_) chamber. Brain extractions and cortex isolation were performed cold on Petri dishes with Hank’s solution (HBSS, Lonza). For tissue dissociation, a previously standardized protocol was followed [62], and subsequently, the cell count was performed in a Neubauer chamber. For cytotoxicity assays, cell suspensions were seeded in plates of 96 wells at a concentration of ~3.5 × 10^4^ cells/well; for the evaluation of the effect of extract and/or inhibitors on dendritic complexity ~1.5 × 10^4^ cells/well were seeded in plates of 24 wells (with 18 mm object covers). The neurons were kept incubated (37 °C and 5% CO_2_) for five days in vitro (DIV) in Neurobasal medium (Gibco) supplemented with 2% B-27 (Gibco) and 0.25% GlutaMax. The respective treatments to the neurons (with *Lippia alba* extract and/or inhibitors) were initiated during DIV6.

### 4.5. Effect of Lippia alba Extract and Specific Inhibitors on the Dendritic Complexity of Cortical Neurons

This experimental protocol was established considering previous results obtained by our research group, which discarded the cytotoxic effect of the *Lippia alba* extract at concentrations of 0.01, 0.03, 0.1, 0.3, and 1.0 μg/mL. The previous results also have shown that the dendritogenic effect of the extract was more potent at the concentrations of 0.01 and 0.1 μg/mL [63].

#### 4.5.1. Treatment with *Lippia alba* Extract and/or Specific Inhibitors

A stock solution of *Lippia alba* (1 mg/mL) was prepared after dissolving it in dimethyl sulfoxide (DMSO), and working dilutions were obtained, ensuring that the final concentration of DMSO was ≤0.2%. To evaluate the kinetics of the dendritogenic effect induced by the *Lippia alba* extract, the supplemental medium was removed from DIV6 neurons and replaced by a mixture of the extract (0.01 μg/mL) and renewed Neurobasal medium. Medium controls (wells to which only Neurobasal was added) and vehicle controls (wells to which a mixture of medium + DMSO at 0.2% were added) were used. The treatment effect was assessed for four time intervals (3 h, 6 h, 12 h, and 24 h). In the protocol to explore the possible involvement of the PI3K and/or MAPK signaling pathways in the dendritogenic effect of the extract, neurons were pretreated for 1 h with specific inhibitors of AKT (PD98059-10 μM) or ERK1/ERK2 (LY294002-10 μM), respectively, and then co-treated with a mixture of the inhibitor (10 μM) + *Lippia alba* extract (0.01 μg/mL) for 6 h. After the respective time intervals of each treatment (both for the kinetics and inhibition protocols), the cells were washed with PBS 1X, and immunocytochemistry was performed. For all experiments, at least four technical replicates (wells/treatment) and two biological replicates (different cell cultures) were performed.

The viability of neurons treated with the inhibitors PD98059 10 μM and LY294002-10 μM (diluted in 0.2% DMSO) was determined at DIV6 by estimating the metabolic activity by reducing MTT—3-(4,5-Bromide dimethylthiazol-2-yl)-2,5-diphenyltetrazolium [64]. Absorbance at 555 nm was determined using a microplate reader (FLUOstar Omega-BMG Labtech, NC, USA). These results were compared with those of two populations of control neurons (some to which only Neurobasal medium and others to which medium + 0.2% DMSO was added). The percentage of cell viability was determined as the ratio between the absorbance of neurons treated with each inhibitor and the absorbance of control neurons.

#### 4.5.2. Immunohistochemistry

This assay was performed by detecting microtubule-associated protein (MAP2), an optimal marker of dendrites in neuronal cultures [65]. It was established to identify changes produced by the plant extract on dendritic complexity at different time intervals and to determine whether these differed when pretreated with ERK1/ERK2 and AKT inhibitors. After the respective treatment and washing with PBS 1X, the cells were fixed by exposing them to 2% paraformaldehyde (diluted in PBS 0.1 M, pH 7.4) for 10 min. Subsequently, they were permeabilized with Triton X-100 at 0.3% (5 min) and blocked with bovine serum albumin (2% BSA diluted in TBS) for 1 h and then incubated with the primary antibody (anti-MAP2 1:5000) overnight at 4 °C. After this time, the cells were rewashed with PBS 1X and incubated with the secondary antibody (Alexa Fluor, 1:1000) for 1 h at room temperature. Finally, the covers were fixed on slide sheets using ProLong Diamond Antifade (Thermo Scientific, Waltham, MA, USA).

#### 4.5.3. Imaging and Processing

Images of cells previously fixed and labeled for MAP2 were obtained by epifluorescence microscopy (Zeiss AxioScope A1) through Zen software (v. lite 2.1), with a 40× objective. The resulting images (in czi format) were processed in Fiji [66] to obtain the 8-bit version in high-resolution format (.tiff). The quantification of changes in the morphological parameters of neurons was performed with the WIS-Neuromath software (v. 3.4.8) [67]. The dendritic complexity of the cells under each treatment was assessed through Sholl’s analysis, a widely used tool for quantifying dendritic arborization under different conditions [68,69,70]. This analysis was carried out through the specialized software SynD (v. 2017.m) [71]. For each treatment or condition, at least 50 images were analyzed, representative of at least two independent biological replicates.

### 4.6. Statistical Analysis

All statistical analyses were performed on GraphPad Prism v. 8.0.2 software (GraphPad Software, San Diego, CA, USA). A one-way ANOVA was used to compare the morphological parameters of the neurons and to contrast the dendritic complexity, a two-way ANOVA was performed. Tukey’s post hoc test was applied for multiple comparisons. For Sholl’s analysis, the calculation was performed on aggregated data (area under the curve). Data are expressed as mean ± standard mean error (SEM). The established significance level was *p* < 0.05 (compared to the control or the vehicle).

## 5. Conclusions

The ethanolic extract of *Lippia alba* leaves showed potential as a psychoplastogen by increasing the branching and complexity of the dendritic tree in rat cortical neurons. The effect was rapid and sustained and seemed to be mediated by the PI3K pathway, which is involved in neuronal survival and proliferation. This finding suggests that the antidepressant effect that has been attributed to the plant could be mediated by inducing structural plasticity, which is affected in mood disorders such as major depression. Future behavioral studies would add important evidence to corroborate the plant’s therapeutic potential.

## Figures and Tables

**Table 1:** Preliminary phytochemical analysis of the extract of *Lippia alba* leaves.

Metabolite Family	Chemical Test	Positive Control	Observation
Steroids	Vanillin-H_2_SO_4_	Lupeol	Negative
Flavonoids	Shinoda	Kaempferol	Positive
Phenolic compounds	FeCl_3_ 1%	Tannic acid	Positive
Coumarins	Fluorescence + NaOH	Coumarin	Negative
Alkaloids	Dragendorff	Theobromine	Negative
Tannins	Lead acetate 10%	Gallic acid	Positive

## Data Availability

The data that support the findings of this study are available from the corresponding author upon request.

## Sample Availability

Samples of the compounds are available from the authors.

## References

[1] Lefebvre J.L., Sanes J.R., Kay J.N., Sickkids J.L. (2015). Development of Dendritic Form and Function. Annu. Rev. Cell. Dev. Biol..

[2] Von Bohlen Und Halbach O. (2013). Analysis of Morphological Changes as a Key Method in Studying Psychiatric Animal Models. Cell. Tissue Res..

[3] O’Neill K.M., Akum B.F., Dhawan S.T., Kwon M., Langhammer C.G., Firestein B.L. (2015). Assessing Effects on Dendritic Arborization Using Novel Sholl Analyses. Front. Cell. Neurosci..

[4] Kulkarni V.A., Firestein B.L. (2012). The Dendritic Tree and Brain Disorders. Mol. Cell. Neurosci..

[5] Forrest M.P., Parnell E., Penzes P. (2018). Dendritic Structural Plasticity and Neuropsychiatric Disease. Nat. Rev. Neurosci..

[6] Bernardinelli Y., Nikonenko I., Muller D. (2014). Structural Plasticity: Mechanisms and Contribution to Developmental Psychiatric Disorders. Front. Neuroanat..

[7] Quach T.T., Stratton H.J., Khanna R., Kolattukudy P.E., Honnorat J., Meyer K., Duchemin A.M. (2020). Intellectual Disability: Dendritic Anomalies and Emerging Genetic Perspectives. Acta Neuropathol..

[8] Qiao H., Li M.X., Xu C., Chen H.B., An S.C., Ma X.M. (2016). Dendritic Spines in Depression: What We Learned from Animal Models. Neural. Plast..

[9] Colyn L., Venzala E., Marco S., Perez-Otaño I., Tordera R.M. (2019). Chronic Social Defeat Stress Induces Sustained Synaptic Structural Changes in the Prefrontal Cortex and Amygdala. Behav. Brain Res..

[10] Wang Y.-T., Wang X.-L., Feng S.-T., Chen N.-H., Wang Z.-Z., Zhang Y. (2021). Novel Rapid-Acting Glutamatergic Modulators: Targeting the Synaptic Plasticity in Depression. Pharmacol. Res..

[11] Culpepper L., Lam R.W., McIntyre R.S. (2017). Cognitive Impairment in Patients with Depression: Awareness, Assessment, and Management. J. Clin. Psychiatry.

[12] Rădulescu I., Drăgoi A.M., Trifu S.C., Cristea M.B. (2021). Neuroplasticity and Depression: Rewiring the Brain’s Networks through Pharmacological Therapy (Review). Exp. Ther. Med..

[13] Yang T., Nie Z., Shu H., Kuang Y., Chen X., Cheng J., Yu S., Liu H. (2020). The Role of BDNF on Neural Plasticity in Depression. Front. Cell. Neurosci..

[14] Gonzalez A., Moya-Alvarado G., Gonzalez-Billaut C., Bronfman F.C. (2016). Cellular and Molecular Mechanisms Regulating Neuronal Growth by Brain-Derived Neurotrophic Factor. Cytoskeleton.

[15] Matsuda S., Ikeda Y., Murakami M., Nakagawa Y., Tsuji A., Kitagishi Y. (2019). Roles of PI3K/AKT/GSK3 Pathway Involved in Psychiatric Illnesses. Diseases.

[16] Huang Y.J., Lane H.Y., Lin C.H. (2017). New Treatment Strategies of Depression: Based on Mechanisms Related to Neuroplasticity. Neural. Plast..

[17] Pizzagalli D.A., Roberts A.C. (2022). Prefrontal Cortex and Depression. Neuropsychopharmacology.

[18] Belleau E.L., Treadway M.T., Pizzagalli D.A. (2019). The Impact of Stress and Major Depressive Disorder on Hippocampal and Medial Prefrontal Cortex Morphology. Biol. Psychiatry.

[19] Johnson M.W., Griffiths R.R. (2017). Potential Therapeutic Effects of Psilocybin. Neurotherapeutics.

[20] Carhart-Harris R.L., Roseman L., Bolstridge M., Demetriou L., Pannekoek J.N., Wall M.B., Tanner M., Kaelen M., McGonigle J., Murphy K. (2017). Psilocybin for Treatment-Resistant Depression: FMRI-Measured Brain Mechanisms. Sci. Rep..

[21] Ly C., Greb A.C., Cameron L.P., Wong J.M., Barragan E.V., Wilson P.C., Burbach K.F., Soltanzadeh Zarandi S., Sood A., Paddy M.R. (2018). Psychedelics Promote Structural and Functional Neural Plasticity. Cell. Rep..

[22] Lee H.M., Roth B.L. (2012). Hallucinogen Actions on Human Brain Revealed. Proc. Natl. Acad. Sci. USA.

[23] Vargas M.V., Meyer R., Avanes A.A., Rus M., Olson D.E. (2021). Psychedelics and Other Psychoplastogens for Treating Mental Illness. Front. Psychiatry.

[24] Bogenschutz M.P., Ross S. (2018). Therapeutic Applications of Classic Hallucinogens. Curr. Top. Behav. Neurosci..

[25] Reiff C.M., Richman E.E., Nemeroff C.B., Carpenter L.L., Widge A.S., Rodriguez C.I., Kalin N.H., McDonald W.M. (2020). Psychedelics and Psychedelic-Assisted Psychotherapy. Am. J. Psychiatry.

[26] de Veen B.T.H., Schellekens A.F.A., Verheij M.M.M., Homberg J.R. (2017). Psilocybin for Treating Substance Use Disorders?. Expert Rev. Neurother..

[27] Johnson M.W., Griffiths R.R., Hendricks P.S., Henningfield J.E. (2018). The Abuse Potential of Medical Psilocybin According to the 8 Factors of the Controlled Substances Act. Neuropharmacology.

[28] Davis A.K., Barrett F.S., May D.G., Cosimano M.P., Sepeda N.D., Johnson M.W., Finan P.H., Griffiths R.R. (2021). Effects of Psilocybin-Assisted Therapy on Major Depressive Disorder: A Randomized Clinical Trial. JAMA Psychiatry.

[29] Nutt D.J., King L.A., Nichols D.E. (2013). Effects of Schedule I Drug Laws on Neuroscience Research and Treatment Innovation. Nat. Rev. Neurosci..

[30] Alvarado-García P.A., Soto-Vásquez M.R., Rosales-Cerquin L.E., Alfaro-Ttito B.M., Rodrigo-Villanueva E.M. (2021). Anxiolytic-like Effect of Essential Oils Extracted from *Lippia alba* and *Lippia citriodora*. Pharmacogn. J..

[31] Da Silva L.V.F., Mouraõ R.H.V., Manimala J., Lnenicka G.A. (2018). The Essential Oil of *Lippia alba* and Its Components Affect Drosophila Behavior and Synaptic Physiology. J. Exp. Biol..

[32] Serafini G. (2012). Neuroplasticity and Major Depression, the Role of Modern Antidepressant Drugs. World J. Psychiatry.

[33] McEwen B.S., Eiland L., Hunter R.G., Miller M.M. (2012). Stress and Anxiety: Structural Plasticity and Epigenetic Regulation as a Consequence of Stress. Neuropharmacology.

[34] Copf T. (2016). Impairments in Dendrite Morphogenesis as Etiology for Neurodevelopmental Disorders and Implications for Therapeutic Treatments. Neurosci. Biobehav. Rev..

[35] Jan Y.N., Jan L.Y. (2010). Branching out: Mechanisms of Dendritic Arborization. Nat. Rev. Neurosci..

[36] Su Y., Liu J., Yu B., Ba R., Zhao C. (2019). Brpf1 Haploinsufficiency Impairs Dendritic Arborization and Spine Formation, Leading to Cognitive Deficits. Front. Cell Neurosci..

[37] Olson D.E. (2018). Psychoplastogens: A Promising Class of Plasticity-Promoting Neurotherapeutics. J. Exp. Neurosci..

[38] Gutiérrez S.L.G., Chilpa R.R., Jaime H.B. (2014). Medicinal Plants for the Treatment of “Nervios”, Anxiety, and Depression in Mexican Traditional Medicine. Rev. Bras. Farmacogn..

[39] Siqueira-Lima P.S., Passos F.R.S., Lucchese A.M., Menezes I.R.A., Coutinho H.D.M., Lima A.A.N., Zengin G., Quintans J.S.S., Quintans-Júnior L.J. (2019). Central Nervous System and Analgesic Profiles of Lippia Genus. Rev. Bras. Farmacogn..

[40] Parente M.S.R., Custódio F.R., Cardoso N.A., Lima M.J.A., Melo T., Linhares M.I., Siqueira R.M.P., Nascimento A., Catunda Júnior F.E.A., Melo C. (2018). Antidepressant-like Effect of *Lippia sidoides* CHAM (Verbenaceae) Essential Oil and Its Major Compound Thymol in Mice. Sci. Pharm..

[41] Haldar S., Kar B., Dolai N., Kumar R.B.S., Behera B., Haldar P.K. (2012). In Vivo Anti-Nociceptive and Anti-Inflammatory Activities of *Lippia alba*. Asian Pac. J. Trop. Dis..

[42] Do Vale T.G., Furtado E.C., Santos J.G., Viana G.S.B. (2002). Central Effects of Citral, Myrcene and Limonene, Constituents of Essential Oil Chemotypes from *Lippia alba* (Mill.) N.E. Brown. Phytomedicine.

[43] Mesa-Arango A.C., Montiel-Ramos J., Zapata B., Durán C., Betancur-Galvis L., Stashenko E. (2009). Citral and Carvone Chemotypes from the Essential Oils of Colombian *Lippia alba* (Mill.) N.E. Brown: Composition, Cytotoxicity and Antifungal Activity. Mem. Inst. Oswaldo Cruz..

[44] Hennebelle T., Sahpaz S., Gressier B., Joseph H., Bailleul F. (2008). Antioxidant and Neurosedative Properties of Polyphenols and Iridoids from *Lippia alba*. Phytother. Res..

[45] Chies C.E., Branco C.S., Scola G., Agostini F., Gower A.E., Salvador M. (2013). Antioxidant Effect of *Lippia alba* (Miller) N. E. Brown. Antioxidants.

[46] Singh B., Sharma R.A. (2015). Plant Terpenes: Defense Responses, Phylogenetic Analysis, Regulation and Clinical Applications. 3 Biotech.

[47] Lima P.S.S., Lucchese A.M., Araújo-Filho H.G., Menezes P.P., Araújo A.A.S., Quintans-Júnior L.J., Quintans J.S.S. (2016). Inclusion of Terpenes in Cyclodextrins: Preparation, Characterization and Pharmacological Approaches. Carbohydr. Polym..

[48] Perez-Vizcaino F., Fraga C.G. (2018). Research Trends in Flavonoids and Health. Arch. Biochem. Biophys..

[49] Stotz S.C., Vriens J., Martyn D., Clardy J., Clapham D.E. (2008). Correction: Citral Sensing by Transient Receptor Potential Channels in Dorsal Root Ganglion Neurons. PLoS ONE.

[50] Quintans L.J., Guimarães A.G., de Santana M.T., Araújo B.E.S., Moreira F.V., Bonjardim L.R., Araújo A.A.S., Siqueira J.S., Ângelo A.R., Botelho M.A. (2011). Citral Reduces Nociceptive and Inflammatory Response in Rodents. Rev. Bras. Farmacogn..

[51] Casadesus G., Shukitt-Hale B., Stellwagen H.M., Zhu X., Lee H.G., Smith M.A., Joseph J.A. (2004). Modulation of Hippocampal Plasticity and Cognitive Behavior by Short-Term Blueberry Supplementation in Aged Rats. Nutr. Neurosci..

[52] Gutierrez-Merino C., Lopez-Sanchez C., Lagoa R., Samhan-Arias A.K., Bueno C., Garcia-Martinez V. (2011). Neuroprotective Actions of Flavonoids. Curr. Med. Chem..

[53] Spencer J.P.E. (2009). The Impact of Flavonoids on Memory: Physiological and Molecular Considerations. Chem. Soc. Rev..

[54] Mansuri M.L., Parihar P., Solanki I., Parihar M.S. (2014). Flavonoids in Modulation of Cell Survival Signalling Pathways. Genes Nutr..

[55] Moosavi F., Hosseini R., Saso L., Firuzi O. (2015). Modulation of Neurotrophic Signaling Pathways by Polyphenols. Drug Des. Devel. Ther..

[56] Numakawa T., Odaka H., Adachi N. (2018). Actions of Brain-Derived Neurotrophin Factor in the Neurogenesis and Neuronal Function, and Its Involvement in the Pathophysiology of Brain Diseases. Int. J. Mol. Sci..

[57] Mullen L.M., Pak K.K., Chavez E., Kondo K., Brand Y., Ryan A.F. (2012). Ras/P38 and PI3K/Akt but Not Mek/Erk Signaling Mediate BDNF-Induced Neurite Formation on Neonatal Cochlear Spiral Ganglion Explants. Brain Res..

[58] Jin Y., Sui H.J., Dong Y., Ding Q., Qu W.H., Yu S.X., Jin Y.X. (2012). Atorvastatin Enhances Neurite Outgrowth in Cortical Neurons in Vitro via Up-Regulating the Akt/MTOR and Akt/GSK-3β Signaling Pathways. Acta Pharmacol. Sin..

[59] Kumar V., Zhang M.-X.X., Swank M.W., Kunz J., Wu G.-Y.Y. (2005). Regulation of Dendritic Morphogenesis by Ras-PI3K-Akt-MTOR and Ras-MAPK Signaling Pathways. J. Neurosci..

[60] Nourbakhsh K., Yadav S. (2021). Kinase Signaling in Dendritic Development and Disease. Front. Cell. Neurosci..

[61] Sanabria-Galindo A., López S.I., Gualdrón R. (1997). Estudio Fitoquímico Preliminar y Letalidad Sobre Artemia Salina de Plantas Colombianas. Rev. Colomb. Cienc. Quím. Farm..

[62] Beaudoin G.M.J., Lee S.H., Singh D., Yuan Y., Ng Y.G., Reichardt L.F., Arikkath J. (2012). Culturing Pyramidal Neurons from the Early Postnatal Mouse Hippocampus and Cortex. Nat. Protoc..

[63] Rodríguez A. (2021). Evaluación Del Efecto Neuroprotector y de Los Cambios en la Complejidad Dendrítica Inducidos por los Extractos de *Tillandsia usneoides* y *Lippia alba* en Cultivo Primario de Neuronas Tratadas Con Agentes Quimioterapéuticos. Master’s Thesis.

[64] Gerlier D., Thomasset N. (1986). Use of MTT Colorimetric Assay to Measure Cell Activation. J. Immunol. Methods.

[65] Korzhevskii D.E., Karpenko M.N., Kirik O.V. (2012). Microtubule-Associated Proteins as Indicators of Differentiation and the Functional State of Nerve Cells. Neurosci. Behav. Physiol..

[66] Schindelin J., Arganda-Carreras I., Frise E., Kaynig V., Longair M., Pietzsch T., Preibisch S., Rueden C., Saalfeld S., Schmid B. (2012). Fiji: An Open-Source Platform for Biological-Image Analysis. Nat. Methods.

[67] Rishal I., Golani O., Rajman M., Costa B., Ben-Yaakov K., Schoenmann Z., Yaron A., Basri R., Fainzilber M., Galun M. (2012). WIS-Neuromath Enables Versatile High Throughput Analyses of Neuronal Processes. Dev. Neurobiol..

[68] Sholl D.A. (1953). Dendritic Organization in the Neurons of the Visual and Motor Cortices of the Cat. J. Anat..

[69] Uylings H.B.M., Pelt J. (2002). van Measures for Quantifying Dendritic Arborizations. Netw. Comput. Neural Syst..

[70] Ristanović D., Milošević N.T., Štulić V. (2006). Application of Modified Sholl Analysis to Neuronal Dendritic Arborization of the Cat Spinal Cord. J. Neurosci. Methods.

[71] Schmitz S.K., Hjorth J.J.J.J., Joemai R.M.S.S., Wijntjes R., Eijgenraam S., de Bruijn P., Georgiou C., de Jong A.P.H.H., van Ooyen A., Verhage M. (2011). Automated Analysis of Neuronal Morphology, Synapse Number and Synaptic Recruitment. J. Neurosci. Methods.

